# Pre-Pregnancy Obesity and Infants’ Motor Development within the First Twelve Months of Life: Who Is Expected to Be the Ultimate Carrier of the Obesity Burden?

**DOI:** 10.3390/nu16091260

**Published:** 2024-04-24

**Authors:** Milan Lackovic, Dejan Nikolic, Biljana Milicic, Dejan Dimitrijevic, Ivona Jovanovic, Sofija Radosavljevic, Sladjana Mihajlovic

**Affiliations:** 1 Department of Obstetrics and Gynecology, University Hospital “Dragisa Misovic”, Heroja Milana Tepica 1, 11000 Belgrade, Serbia; lackovic011@gmail.com; 2 Faculty of Medicine, University of Belgrade, 11000 Belgrade, Serbia; denikol27@gmail.com (D.N.); dimitrijevic.dejan@gakfront.org (D.D.); 3 Department of Physical Medicine and Rehabilitation, University Children’s Hospital, 11000 Belgrade, Serbia; 4 Department of Medical Statistics, Faculty of Dental Medicine, University of Belgrade, 11000 Belgrade, Serbia; 5 Clinic for Gynecology and Obstetrics “Narodni Front”, 11000 Belgrade, Serbia; ivona.vuceljic@gmail.com; 6 Department of Radiology, University Hospital “Dragisa Misovic”, Heroja Milana Tepica 1, 11000 Belgrade, Serbia

**Keywords:** pre-pregnancy obesity, offspring, early motor development, complications

## Abstract

Introduction: Pre-pregnancy obesity is a significant public health concern with profound implications for maternal and child health. The burgeoning evidence suggests that maternal obesity prior to conception is intricately linked with an increased risk of gestational complications, as well as with adverse neonatal outcomes. Furthermore, the long and short-term health of offspring, including the risk of early motor development impairment, obesity, and metabolic syndrome in childhood and adulthood, may be adversely affected as well. Addressing pre-pregnancy obesity is critical for improving overall maternal and child health outcomes, and therefore, the aim of this study was to evaluate the connections linking pre-pregnancy obesity with infants’ motor development within the first twelve months of infants’ lives. Material and Methods: This study included 200 mother–infant pairs divided into two groups based on their pre-pregnancy body mass index values. To assess infants’ early motor development, we used the Alberta Infant Motor Scale (AIMS) and evaluated the parameters of infants’ early motor development at the ages of three, six, nine, and twelve months. Results: Pre-pregnancy overweight/obesity was significantly associated with excessive gestational weight gain (*p* < 0.001), fetal macrosomia (*p* = 0.022), and a family history of diabetes and cardiovascular diseases (*p* = 0.048 and *p* = 0.041, respectively), as well as with all observed parameters of early motor development at the ages of three, six, nine, and twelve months: AIMS 3 months total (*p* < 0.001), AIMS 6 months total (*p* < 0.001), AIMS 9 months total (*p* < 0.001), and AIMS 12 months total (*p* < 0.001). Furthermore, pre-pregnancy overweight/obesity was a significant predictor for AIMS 6 months total (*p* = 0.043) and AIMS 6 months supination (*p* = 0.017). Conclusions: Pre-pregnancy obesity is a critical determinant of pregnancy outcomes and offspring early motor development, with possible far-reaching implications for children’s long-term well-being. Addressing this issue requires a comprehensive approach that includes preconception weight management, targeted interventions during the pregnancy and postpartum periods, and ongoing research to better understand the underlying mechanisms and develop effective strategies for prevention and management.

## 1. Introduction

The exponential growth rate of overweight and obesity over the past few decades has reached epidemic proportions globally, with a significant percentage of women of childbearing age being affected. The reproductive consequences of obesity are numerous, from menstrual cycle disorders and fertility difficulties to potentially life threatening conditions associated with pregnancy and the postpartum period [1]. Pre-pregnancy obesity is a significant public health concern with profound implications for maternal and child health [2]. Burgeoning evidence suggests that maternal obesity prior to conception is intricately linked with an increased risk of gestational complications, as well as with complications extending far beyond pregnancy, affecting the life-long health of the offspring [3]. Pregnant women dealing with overweight and obesity are at an increased risk of early pregnancy losses, congenital malformations, the delivery of macrosomic infants, shoulder dystocia, stillbirths, spontaneous or medically induced preterm deliveries, as well as with all prematurity related complications and consequences [4]. Hypertensive disorder of pregnancy (HDP), one of the leading causes of maternal mortality in developed countries, is closely associated with maternal obesity. HDP may lead to severe maternal morbidity, including peripartum heart attack and stroke, and it carries a risk of early cardiovascular disease onset and a life-long risk of metabolic, cerebral, and renal diseases [5,6]. 

The underlying mechanisms linking pre-pregnancy obesity to adverse outcomes are complex and multifaceted. Insulin resistance, altered nutrient supply, chronic low-grade inflammation, and hormonal imbalances are the key factors that contribute to the development of gestational complications, and exposure to an obesogenic intrauterine environment may predispose offspring to metabolic disorders, immune dysregulation, and altered neurodevelopment [2,3,7]. The Barker Hypothesis, or the Developmental Origins of Health and Disease (DOHaD) theory, supports this, suggesting that the intrauterine environment can have long-lasting effects on gene expression and health outcomes [8]. Heritable changes in gene expression that occur without alterations in the deoxyribonucleic acid (DNA) sequence itself are in the essence of epigenetics, and such changes may be induced by environmental factors, including maternal obesity and excessive body fat accumulation. Furthermore, exposure to maternal obesity can affect the development of neural circuits in the fetus that regulate appetite and energy balance, predisposing the offspring to obesity and related disorders [9].

During the first year of life, infants experience rapid growth and development, especially in their gross motor skills. Gross motor development refers to the progression of physical abilities in infants and children, such as gaining head control, rolling over, sitting, crawling, standing, and later, walking and running. These skills require the involvement of the large muscles of the body, and there is growing academic interest in understanding how various factors, including maternal obesity, might influence infants’ motor development. Gross motor development typically follows predictable sequences and patterns, though the exact timing can vary from one infant to another, and each milestone reflects an infant’s increasing muscle strength, coordination, and neural development [10]. 

Maternal obesity can influence infants’ motor development in several different ways, potentially leading to delays or alterations in achieving early motor milestones. Recent research findings suggest that children born to mothers who are overweight or obese may be at an increased risk of delayed gross motor development [9,11,12]. The mechanisms and reasons for these influences are multifaceted and can include a combination of genetic, environmental, and intrauterine factors. Aside from pregnancy-related complications and an altered intrauterine environment, maternal obesity may be closely related to a higher incidence of difficult deliveries and birth complications, which may temporarily or permanently impair an infant’s motor function. The postnatal environment, including parenting practices, physical activity levels, and socioeconomic status, can also influence motor development. Infants of mothers with obesity may have different experiences in these areas, which can impact their motor skill development. For instance, if maternal obesity limits a mother’s physical activity level, the infant may have fewer opportunities for active play, which is crucial for developing motor skills. As previously discussed, genetic predispositions remain another important concern, since epigenetic modifications resulting from maternal obesity may influence the neurodevelopment of the child, including the performance of their motor skills [9,11,12,13,14,15].

Measuring infants’ early motor development involves assessing their progress in acquiring motor skills, which are critical indicators of their overall health and development. There are several standardized tools and observational methods used by pediatricians, researchers, and therapists to evaluate motor development in infants. These assessments can help identify potential delays or difficulties early on, allowing for timely interventions. The Bayley Scales of Infant and Toddler Development, Ages and Stages Questionnaires, Denver Developmental Screening Test, Peabody Developmental Motor Scales, and Alberta Infant Motor Scale are some of the most frequently used assessment tools to quantify infants’ early motor progression [16]. The Alberta Infant Motor Scale (AIMS) is an observational assessment specifically designed to evaluate the gross motor maturation of infants from birth until the independent walking stage. It observes spontaneous movements in four positions, namely prone (on the stomach), supine (on the back), sitting, and standing, and it helps in identifying infants who are delayed in their gross motor development. The AIMS was validated in Serbian infants with high consistency, reliability, and temporal stability [17]. All of these assessment tools were designed to be used by trained professionals and require interpretation within the context of each child’s overall development and environmental factors. The early identification of motor development issues is extremely important as it can lead to early intervention services, which are critical for improving outcomes among children who are born at risk for early motor impairment.

The increasing prevalence of pre-pregnancy obesity underscores the urgent need to understand its impact and develop effective strategies to mitigate its effects. Year after year, unstoppable growth in the prevalence of children born to mothers with obesity is observed, with a possibility of the onset of a whole new spectrum of concerns and disorders that might affect infants’ neurodevelopment and lead to consequences for the offspring. Therefore, the aim of our study was to address pre-pregnancy obesity and its short-term effect on infants’ early motor development during the first twelve months of infants’ lives.

## 2. Methods

### 2.1. Study Design and Participants

This study took place in the university hospital named after “Dr. Dragisa Misovic” in Belgrade, Serbia. Participants were randomly selected from the computer database at the obstetric ward of the hospital where delivery was performed based on maternal weight. Every 5th woman admitted to the hospital for delivery was selected up to the number 200.

The selection of participants lasted between 2019 and 2020. Two hundred “mother–infant” pairs were included, and the study was conducted as a clinical observational study. Principles of the Declaration of Helsinki and good clinical practice were applied, and approval was obtained from the hospital’s Institutional Review Board (IRB) (No. 01-14706/19, Date: 22 November 2019).

Study participants were divided into two groups based on their body mass index (BMI) values and the World Health Organization’s (WHO) recommendations. The study group consisted of 88 subjects whose pre-pregnacy weights were beyond the reference range for normal weight according to the WHO’s recommendations, and the control group was composed of 112 subjects whose BMI values were within the WHO range for normal weight. All participants were advised to take 400 micrograms of folic acid during the first twelve weeks of pregnancy as well as pregnancy supplements. Additional nutritional counselling was not provided, and the gestations were carried out with their former habits from the pre-pregnancy period. 

According to the BMI values, calculated as weight in kilograms divided by height in meters squared (kg/m^2^), and the WHO recommendations, four nutritional statuses were defined: underweight (BMI < 18.5 kg/m^2^), normal weight (BMI between 18.5 and 24.9 kg/m^2^), overweight (BMI between 25.0 and 29.9 kg/m^2^), and obese (BMI > 30.0 kg/m^2^) [18].

### 2.2. Study Exclusion Criteria

Maternal age less than 18 and more than 45 at the time of conception, multiple pregnancies, and chronic health disorders were defined as exclusion criteria. Fetal and newborn malformations or defects, the presence of stigmata during the examination of neonates (dyscrania, hypertrichosis, asymmetry of the gluteal furrow, and fovea spinalis), hydrocephalus, atrhrogryposis, open spinal dysraphism, congenital rigid equinovarus, and aplasia of the bones of the lower and upper extremities were the exclusion criteria for the follow-up of the newborns from the study.

### 2.3. Study Variables

Data on family history of diabetes mellitus (DM) and cardiovascular disease (CVD) were collected from patients’ primary health reports. Gestational weight gain was calculated as the mathematical difference between participants’ weights before conception and weight at delivery and classified as excessive based on the Institute of Medicine’s (IOM) recommendations. Weight gain beyond 18.0 kg for underweight, 16.0 kg for normal weight, 11.5 kg for overweight, and 9.0 kg for obesity were defined as excessive according to the IOM guidelines [19]. Birth weight over 4000 g was diagnostic criterion for fetal macrosomia [20].

The Alberta Infant Motor Scale (AIMS) was used to asses infants’ early motor development. This scale consists of 58 domains, including pronation (21), supination (9), sitting (12), and standing (16), and it is a non-referenced measure with high specificity and sensitivity [11]. A trained assistant physician under the supervision of a specialist in physical medicine and rehabilitation performed all AIMS measurements.

### 2.4. Statistical Analysis 

The Statistical Package for Social Science (SPSS Statistics V.22.0) was used for all of the data analyses. Comparisons between the examined patient groups were performed using the Mann–Whitney U test for continuous variables and the chi-squared test for categorical variables. Univariate and multivariable binary logistic regression methods were used to analyze the association between observed parameters and pre-pregnancy overnutrition. Differences were considered significant when the *p* value was <0.05.

## 3. Results

The patients with increased BMI values had significantly more frequent EGWG (*p* < 0.001), a positive family history of CVD (*p* = 0.014), a positive family history of DM (*p* = 0.042), and fetal macrosomia (*p* = 0.007) (Table 1). The newborns from mothers with increased BMI values had significantly lower values for AIMS at 3 months for pronation and supination and in total (*p* < 0.001). Regarding the age of 6 months, the AIMS values were significantly lower in the newborns from mothers with increased BMI values for pronation and supination (*p* < 0.001), sitting (*p* = 0.021), and in total (*p* < 0.001). At the age of 9 months, significantly lower AIMS values were noticed for pronation, sitting, standing, and in total (*p* < 0.001), as well for supination (*p* = 0.002). Regarding the age of 12 months, the AIMS values were significantly lower in the newborns from mothers with increased BMI values for sitting (*p* = 0.019), for standing, and in total (*p* < 0.001) (Table 1).

In the univariate logistic regression analysis, pre-pregnancy overweight/obesity was a significant risk factor for excessive gestational weight gain (*p* < 0.001), fetal macrosomia (*p* = 0.022), a family history of cardiovascular disease and diabetes mellitus (*p* = 0.041 and *p* = 0.048, respectively), AIMS pronation at 3 months (*p* < 0.001), AIMS supination at 3 months (*p* < 0.001), AIMS total score at 3 months (*p* < 0.001), AIMS pronation at 6 months (*p* < 0.001), AIMS supination at 6 months (*p* < 0.001), AIMS sitting at 6 months (*p* = 0.028), AIMS total score at 6 months (*p* < 0.001), AIMS pronation at 9 months (*p* < 0.001), AIMS supination at 9 months (*p* < 0.001), AIMS sitting at 9 months (*p* < 0.001), AIMS standing at 9 months (*p* < 0.001), AIMS total score at 9 months (*p* < 0.001), AIMS pronation at 12 months (*p* = 0.004), AIMS sitting at 12 months (*p* < 0.001), AIMS standing at 12 months (*p* < 0.001), and AIMS total score at 12 months (*p* < 0.001) (Table 2).

In the multivariate logistic regression analysis, pre-pregnancy overweight/obesity was a significant risk factor for excessive gestational weight gain (*p* < 0.001), AIMS supination at 6 months (*p* = 0.017), and the AIMS total score at 6 months (*p* = 0.043) (Table 3).

## 4. Discussion

In our study, pre-pregnancy obesity was a risk factor for all observed parameters of early motor development; the data are undoubtedly indicative that infants born to mothers who are overweight and obese are at an increased risk of early gross motor impairment. The only exception observed in our AIMS metrics, where we did not find any difference, was for the AIMS 6 standing. However, at this age, infants are not expected to acquire such skills, and therefore, the lack in differences between the groups is not relevant. 

At the age of about six months, the first major differences in infants’ gross motor skills may appear, since this is the age when infants are usually expected to make some of the more control-demanding postures, including rolling over and sitting up without support [21]. These significant milestones of motor development are therefore of great importance, and the timely recognition of these usually subtle differences is critical for early intervention. Sitting delays have been recognized and well studied as unfavorable outcomes of some pregnancy-related complications, including prematurity [22], and the delayed acquisition of motor skills at this age is known to be associated with early childhood obesity [23]. Epigenetic modifications of neurotrophic genes, maternal and fetal inflammation, alterations to the microbiome, and impaired neurotransmitter signaling are some of the proposed mechanisms linking maternal overnutrition with children’s neurodevelopmental vulnerability [24]. Wylie et al. studied the association between a compromised intrauterine environment and maternal pre-pregnancy obesity and identified maternal nutrition status as a risk factor for infants’ sitting performance [25]. Taveras et al. found an association between infants’ weight-to-length increase during the first six months of life and childhood obesity at the age of three years, and stressed the importance of close infant surveillance during the first six months of an infant’s life [26]. Furthermore, in a recently published study, Aoyama et al. revealed that achieving later gross motor milestones at this age may in fact predict increased body fat for children aged 6 to 7 years [27] and anticipated that maternal pre-pregnancy overnutrition leading to early motor delay may ultimately result in childhood obesity.

Even though there are inconsistencies in the literature, and the interpretation of results linking maternal obesity with children’s neurodevelopment may be challenging primarily due to the amplification of other pre-pregnancy and pregnancy-driven disorders, pregnancy overweight and obesity are recognized as independent risk factors for offspring neurodevelopmental delay [28]. Unlike maternal diabetes and hypertension, maternal preconception BMI trajectories are independently associated with poorer childhood neurodevelopment [29]. Based on the above, the age of six months appears to be a cross row that is not only indicative of our parental legacy, but also of our future perspectives and potentials. The importance of an early quantitative assessment of an infant’s motor development was recognized in a study conducted by Gajewska et al. as well [30]. This group of authors found that infants facing major developmental disorders may be identified and selected for further follow-ups in as early as the age of three months [30]. However, Gajewska et al.’s primary focus of study was to identify infants who are at an increased risk of cerebral palsy, and therefore, we believe that the quantitative assessment of infants’ motor development at the age of six months is more appropriate for more subtle motor impairments, like those associated with maternal obesity. 

Fetal predisposition and susceptibility to gross motor impairment and the offspring phenotype appears to be determined through epigenetic adaptations and placental structural and functional changes, which are associated with maternal obesity [31]. DNA methylation, histone modifications, and microRNAs are epigenetic modifications which may play key roles in disease susceptibility through maternal metabolic alterations [32] and altered neurodevelopment trajectories in offspring [33]. Rapid and excessive weight gain during pregnancy and excess fetal lipid accumulation lead to fetal fat mass increase, accelerated fetal growth dynamics, and fetal macrosomia [34]. An association between maternal obesity and fetal overgrowth resulting in fetal macrosomia appears to be significant [35], and our study results support such association. Furthermore, Zhang et al. found that fetal macrosomia and altered fetal metabolic programming decreases infants’ early adaptability and gross and fine motor performances at the age one to six months [36].

Acknowledgement and early clinical interventions often play crucial roles in supporting infants who show delays or atypical patterns in gross motor development, and they can vary widely based on a child’s individual needs [37]. The effectiveness of clinical interventions can significantly impact infants’ gross motor development, and positive outcomes can include improved muscle strength and coordination, an enhanced ability to reach developmental milestones within a typical age range, increased independence in mobility, and positive effects on social, cognitive, and emotional development, as physical abilities allow for more interactions with the environment and others [38,39]. It is important to note that outcomes can vary based on the nature and timing of the intervention, the specific needs of the child, and the involvement of the family members [37]. Thus, the continuous monitoring and adjustment of interventions are often necessary to meet the evolving needs of the growing infant, and focus should be centered on elucidating the matter of overnutrition, including preconception weight management, nutritional counselling, and regular physical activity, which can significantly improve outcomes for both the mother and the offspring [40,41,42].

Despite pregnancy being the optimal time for adapting healthy lifestyle choices, preconceptionally overweight and obese women unfortunately rarely meet the proposed weight gain goals [43,44]. Women in our study who were overweight and obese tended to gain weight excessively during pregnancy significantly more frequently compared to women with normal weights. Such unappealing tendency, primarily associated with maternal habitual dietary intake patterns, is a common observation reported by researchers studying different populations and ethnicities [45,46]. Achieving optimal gestational weight gain for women who are overweight or obese before pregnancy is crucial for minimizing health risks for both the mother and the fetus. The pattern of gestational weight gain among women dealing with overnutrition is considered as an important marker for fetal growth dynamics, especially during the first 20 weeks of gestation. Maternal obesity is associated with an increased risk for both large-for-gestational-age (LGA) and small-for-gestational-age (SGA) babies, and disentangling gestational weight gain patterns may be helpful in understanding competing risks between LGA and SGA. Among women who are overweight and obese, excessive gestational weight gain before the 20th gestational week is related to LGA regardless of later gain, while lower gestational weight gain during the same gestational age is related with an increased SGA risk [47]. Moreover, excessive gestational weight gain is an independent risk factor for early motor development impairment [48], but it may also have a synergetic effect with pregestational overnutrition [49,50]. Appropriate nutritional intake, regular weight gain monitoring, and interventions conducted early during pregnancy, including diet and physical activity, may have a profound impact on weight gain reduction and comorbidity prevalence for both the mother and the child [51]. 

Another interesting observation of our study’s results is the assumed transgenerational obesity susceptibility. Family history of DM and CVD was more commonly positive in the group of subjects who were overweight and obese compared to their peers with normal weights. Obesity is a major risk factor for both CVD and DM [52], and this result may in fact presume that obesity may act as a villain keeping three generations in captivity and endangering the most valuable resource each living being has to offer, which is healthy offspring. Despite the lack of conclusive evidence, a growing body of research, including systemic reviews, links maternal overnutrition with offspring’s risk of CVD, DM, and even cancer [53]. Nutritional interventions conducted during pregnancy have shown efficiency and beneficial effects on glucose tolerance, insulin resistance, vascular function, and lipid concentrations in offspring, and thereby in decreasing the risk of non-communicable diseases (NCDs) [54]. NCDs, including CVD and type 2 DM, are the leading causes of death and disability worldwide, with a growing incidence year after year. More than half a billion people globally are living with DM and its devastating complications, and it is estimated that there will be an increase of more than double in the next 30 years, resulting in 1.3 billion people being affected [55]. Therefore, this is the time to engage all of our resources and efforts to achieve the prevention of NCDs. Preventing NCDs must start preconceptionally, with targeted intervention and strategies aimed at generations that are in control over their own health and future, as well as of the health and future of their offspring.

There are several limitations to this study. First of all, the study’s participants belong to a Serbian population, and specific socio-economic variations, as well as inherited predispositions, may exist in other populations and ethnicities. The sample size of this study is another limiting factor, since the inclusion of a larger group of participants could increase the sensitivity of the study’s results; thus, additional studies conducted on larger samples are advised. 

## 5. Conclusions

Pre-pregnancy obesity is a critical determinant of pregnancy outcomes and offspring health, with far-reaching implications for the short-term and, possibly, long-term well-being of both the mother and child. Excessive gestational weight gain, which is more prevalent among overweight and obese women, exacerbates the risk of negative outcomes, including impaired motor development, but factors such as genetic predispositions, the postnatal environment, and socioeconomic status may also play critical roles in shaping infants’ developmental trajectories. Therefore, it is essential for medical professionals to incorporate strategies aimed at optimizing maternal weights before and during pregnancy as part of a comprehensive approach to improve infants’ health outcomes. Future research should focus on elucidating the precise biological mechanisms at play, as well as on developing targeted interventions that can mitigate the impact of maternal obesity on child neurodevelopment. Ultimately, addressing maternal obesity represents a critical level for enhancing the developmental prospects of the next generation, underscoring the importance of public health initiatives and clinical practices that promote maternal and infant health.

## Figures and Tables

**Table 1:** Characteristics of tested variables with regard to maternal pre-pregnancy weight status.

Variables		Maternal Pre-Pregnancy Weight Status N = 200		*p*
		Normal Weight	Overweight/Obese	
EGWG N (%)	No	100 (89.3%)	13 (14.8%)	<0.001 *
	Yes	12 (10.7%)	75 (85.2%)	
Family history of CVD N (%)	No	96 (85.7%)	63 (71.6%)	0.014 *
	Yes	16 (14.3%)	25 (28.4%)	
Family history of DM N (%)	No	106 (94.6%)	76 (86.4%)	0.042 *
	Yes	6 (5.4%)	12 (13.6%)	
Fetal macrosomia N (%)	No	101 (90.2%)	67 (76.1%)	0.007 *
	Yes	11 (9.8%)	21 (23.9%)	
AIMS score				
AIMS 3 months (MV ± SD)	Pronation	2.72 ± 0.47	2.42 ± 0.60	<0.001 **
	Supination	2.84 ± 0.37	2.42 ± 0.62	<0.001 **
	Total	5.57 ± 0.72	4.84 ± 1.02	<0.001 **
AIMS 6 months (MV ± SD)	Pronation	15.72 ± 0.47	15.34 ± 0.68	<0.001 **
	Supination	8.77 ± 0.50	8.25 ± 0.87	<0.001 **
	Sitting	6.78 ± 0.64	6.58 ± 0.83	0.021 **
	Standing	1.97 ± 0.34	1.90 ± 0.34	0.125 **
	Total	33.24 ± 1.54	32.07 ± 2.07	<0.001 **
AIMS 9 months (MV ± SD)	Pronation	19.37 ± 0.68	18.93 ± 0.76	<0.001 **
	Supination	8.97 ± 0.16	8.85 ± 0.36	0.002 **
	Sitting	10.21 ± 0.85	9.58 ± 0.99	<0.001 **
	Standing	4.43 ± 0.65	3.94 ± 0.75	<0.001 **
	Total	42.97 ± 2.00	41.31 ± 2.41	<0.001 **
AIMS 12 months (MV ± SD)	Pronation	21.00 ± 0.00	20.99 ± 0.11	0.259 **
	Supination	9.00 ± 0.00	9.00 ± 0.00	1.000 **
	Sitting	11.96 ± 0.21	11.83 ± 0.46	0.019 **
	Standing	15.67 ± 0.62	15.20 ± 0.79	<0.001 **
	Total	57.63 ± 0.75	57.02 ± 1.11	<0.001 **

EGWG—excessive gestational weight gain; CVD—cardiovascular disease; DM—diabetes mellitus; AIMS—Alberta Infant Motor Scale. * Chi-squared test; ** Mann–Whitney U test.

**Table 2:** Univariate logistic regression analysis of tested variables regarding pre-pregnancy overweight/obesity and normal weight.

Variables	Univariate Logistic Regression Analysis (Pre-Pregnancy Overweight/Obesity and Normal Weight)		
	B	95% CI	*p*
**EGWG**	5.318	4.544–6.092	<0.001
**Fetal macrosomia**	1.683	0.249–3.117	0.022
**Family history of CVD**	1.360	0.055–2.666	0.041
**Family history of DM**	1.857	0.013–3.700	0.048
**AIMS pronation 3 months**	−1.919	−2.851–(−)0.988	<0.001
**AIMS supination 3 months**	−3.046	−3.946–(−)2.146	<0.001
**AIMS total score 3 months**	−1.698	−2.218–(−)1.178	<0.001
**AIMS pronation 6 months**	−2.179	−3.017–(−)1.342	<0.001
**AIMS supination 6 months**	−1.934	−2.607–(−)1.260	<0.001
**AIMS sitting 6 months**	−0.808	−1.528–(−)0.088	0.028
**AIMS standing 6 months**	−1.093	−2.647–0.461	0.167
**AIMS total score 6 months**	−0.676	−0.943–(−)0.408	<0.001
**AIMS pronation 9 months**	−1.631	−2.309–(−)0.953	<0.001
**AIMS supination 9 months**	−3.860	−5.748–(−)1.972	<0.001
**AIMS sitting 9 months**	−1.517	−2.028–(−)1.005	<0.001
**AIMS standing 9 months**	−1.932	−2.605–(−)1.258	<0.001
**AIMS total score 9 months**	−0.669	−0.877–(−)0.460	<0.001
**AIMS pronation 12 months**	−10.799	−18.199–(−)3.399	0.004
**AIMS supination 12 months**	-	-	-
**AIMS sitting 12 months**	−2.667	−4.158–(−)1.175	<0.001
**AIMS standing 12 months**	−1.940	−2.613–(−)1.267	<0.001
**AIMS total score 12 months**	−1.509	−2.016–(−)1.002	<0.001

EGWG—Excessive Gestational Weight Gain; CVD—Cardiovascular Disease; DM—Diabetes Mellitus; AIMS—Alberta Infant Motor Scale; B—Beta Coefficient; CI—Confidence Interval.

**Table 3:** Multivariate logistic regression analysis of tested variables regarding pre-pregnancy overweight/obesity and normal weight.

Variables	Multivariate Logistic Regression Analysis (Pre-Pregnancy Overweight/Obesity and Normal Weight)		
	B	95% CI	*p*
**EGWG**	−2.485	−3.170–(−)1.800	<0.001
**Fetal macrosomia**	−0.007	−0.457–0.443	0.974
**Family history of CVD**	0.173	−0.340–0.686	0.506
**Family history of DM**	−0.673	−1.431–0.084	0.081
**AIMS pronation 3 months**	1.148	−1.343–3.638	0.364
**AIMS supination 3 months**	1.485	−0.747–3.717	0.191
**AIMS total score 3 months**	−1.224	−3.360–0.913	0.260
**AIMS pronation 6 months**	−0.266	−1.631–1.100	0.702
**AIMS supination 6 months**	−0.896	−1.631–(−)0.162	0.017
**AIMS sitting 6 months**	−0.462	−1.119–0.195	0.167
**AIMS total score 6 months**	0.616	0.021–1.212	0.043
**AIMS pronation 12 months**	−0.289	−0.633–0.056	0.100
**AIMS supination 9 months**	0.036	−0.764–0.836	0.930
**AIMS sitting 9 months**	0.216	−0.104–0.536	0.185
**AIMS standing 9 months**	−0.280	−0.718–0.157	0.207
**AIMS supination 12 months**	0.238	−2.389–2.865	0.858
**AIMS sitting 12 months**	−0.250	−1.193–0.693	0.601
**AIMS total score 12 months**	−0.305	−0.716–0.105	0.143

EGWG—Excessive Gestational Weight Gain; CVD—Cardiovascular Disease; DM—Diabetes Mellitus; AIMS—Alberta Infant Motor Scale; B—Beta Coefficient; CI—Confidence Interval.

## Data Availability

All data are presented in this study. Original data are available upon reasonable request from the corresponding author.
